# Interactive Effects of Nitrogen and Phosphorus on Soil Microbial Communities in a Tropical Forest

**DOI:** 10.1371/journal.pone.0061188

**Published:** 2013-04-12

**Authors:** Lei Liu, Tao Zhang, Frank S. Gilliam, Per Gundersen, Wei Zhang, Hao Chen, Jiangming Mo

**Affiliations:** 1 Key Laboratory of Vegetation Restoration and Management of Degraded Ecosystems, South China Botanical Garden, Chinese Academy of Sciences, Guangzhou, China; 2 State Key Laboratory of Urban and Regional Ecology, Research Center for Eco-Environmental Sciences, Chinese Academy of Sciences, Beijing, China; 3 Department of Biological Science, Marshall University, Huntington, West Virginia, United States of America; 4 Danish Centre for Forest, Landscape and Planning, University of Copenhagen, Copenhagen, Denmark; DOE Pacific Northwest National Laboratory, United States of America

## Abstract

Elevated nitrogen (N) deposition in humid tropical regions may exacerbate phosphorus (P) deficiency in forests on highly weathered soils. However, it is not clear how P availability affects soil microbes and soil carbon (C), or how P processes interact with N deposition in tropical forests. We examined the effects of N and P additions on soil microbes and soil C pools in a N-saturated old-growth tropical forest in southern China to test the hypotheses that (1) N and P addition will have opposing effects on soil microbial biomass and activity, (2) N and P addition will alter the composition of the microbial community, (3) the addition of N and P will have interactive effects on soil microbes and (4) addition-mediated changes in microbial communities would feed back on soil C pools. Phospholipid fatty acid (PLFA) analysis was used to quantify the soil microbial community following four treatments: Control, N addition (15 g N m^−2^ yr^−1^), P addition (15 g P m^−2^ yr^−1^), and N&P addition (15 g N m^−2^ yr^−1^ plus 15 g P m^−2^ yr^−1^). These were applied from 2007 to 2011. Whereas additions of P increased soil microbial biomass, additions of N reduced soil microbial biomass. These effects, however, were transient, disappearing over longer periods. Moreover, N additions significantly increased relative abundance of fungal PLFAs and P additions significantly increased relative abundance of arbuscular mycorrhizal (AM) fungi PLFAs. Nitrogen addition had a negative effect on light fraction C, but no effect on heavy fraction C and total soil C. In contrast, P addition significantly decreased both light fraction C and total soil C. However, there were no interactions between N addition and P addition on soil microbes. Our results suggest that these nutrients are not co-limiting, and that P rather than N is limiting in this tropical forest.

## Introduction

Biogeochemical cycling of nitrogen (N) is greatly altered by anthropogenic activities, with global cycling rates estimated to have increased by approximately 100% since mid-1900s [1]. In Asia, emissions of reactive N have increased dramatically [2], leading to deposition of 30–73 kg N ha^−1^ yr^−1^ in some sub-tropical forests of southern China [3]. Such high rates of N deposition are comparable to the highest levels of N deposition occurring in forests of North America and Europe [4], [5] where adverse effects of excess N, including soil acidification, nutrient imbalance, nitrate leaching, loss of biodiversity, and even forest decline, have been reported in some forests [6]–[9].

In contrast to temperate forests, which are often N-limited under natural conditions, tropical forests more typically exhibit phosphorus (P) limitation, with soils often highly acidic and low in base cation availability [10], [11]. Most fertilization experiments conducted in tropical forests have demonstrated a stronger response of plant biomass to added P than to added N, supporting the paradigm that tropical ecosystems on old soils are predominantly P limited [1], [12]. Cleveland *et al*. (2011) conducted a meta-analysis of carbon (C)-nutrient-climate relationships in 113 sites across the tropical forest biome and found that P availability regulated net primary production and other ecosystem processes in lowland tropical forests [13]. In contrast to these long-standing ideas, however, recent studies have demonstrated that N, P and K or base cations all limit forest plants growth in tropical forests [14], [15].

Elevated atmospheric N deposition may further enhance such P limitation in tropical forests. Some tropical forests of the high deposition region in southeastern China are indeed N saturated and exhibit high rates of nitrate leaching [3], particularly an old-growth forest of the Dinghushan Biosphere Reserve (DHSBR), which supports representative natural vegetation in the region and receives high N deposition. We have reported that up to 60 kg N ha^−1^yr^−1^ is leached from the old-growth forest, and that Al^3+^ concentrations increased and the soil pH decreased significantly in high N addition plots [16], [17], [18]. Further work demonstrated that the herbaceous layer at this site is quite sensitive to N deposition [19]. A 30-yr time series of plant chemistry and production in this forest revealed signs of progressive P limitation, including decreases in available soil P, increases in N/P ratios in leaves and litterfall and in litterfall amount, and decreases in aboveground primary production [20]. Another study in this forest indicated an increase in the soil organic carbon (SOC) over the same period [21].

The occurrence of opposite trends in plant production and in SOC stock at DHSBR may only be explained by major changes in soil organic matter (SOM) quality and turnover, i.e., that increased N availability and decreased P availability have reduced the decomposition rate of SOM. Although work at DHSBR demonstrated decreases in litter decomposition, soil respiration, and soil microbial biomass with experimental N addition [22]–[25], less is known about the effects of P and particularly about N/P interactions on these processes.

Soil microorganisms are the drivers of decomposition processes, but soil nutrient availability may influence soil microbial growth and activity [26]. Nutritional constraints linked to scarcity of available P can shape soil microbial community composition in highly weathered soils [27]. Gallardo and Schlesinger (1994) suggested that microbial P limitation may be common in highly weathered soils in which P tends to be bound in iron or aluminum sesquioxides [28]. Experimental P additions in tropical rain forests have revealed that microbial utilization of at least labile fractions of soil organic carbon (SOC) was P limited [29]. Moreover, N- and P-induced shifts in microbial communities should cause corresponding shifts in the functional and metabolic potentials of the communities, resulting in a change in decomposition rates [30]. Recently, Cusack *et al*. (2011a) reported links between microbial responses to N deposition and shifts in SOM quality and quantity in two tropical forests [31].

As we known, understanding nutrient limitation is a key to predicting how the C cycle will respond to environmental change [32]. However, identifying nutrient limitation in tropical forests and resolving its importance therein is a complex undertaking. Especially, the effects of nutrient interactions on C cycle in tropical forests are poorly known. Understanding of the effects of N and P as well as their interactions on C processes and soil microbial communities in tropical forests, we established a full factorial N and P addition experiment at DHSBR in 2007 from which some short-term results for the P addition was presented by Liu *et al*. [33].

In this paper we report the long-term (>4 year) changes in microbial biomass, activity and community structure to the N-P treatments. The purpose of this study is to explore potential feedback mechanisms and microbial shifts due to N-P interactions that may explain why SOC accumulates at DHSBR. Our hypotheses are that:i) N and P addition will have opposite effects (decrease and increase, respectively) on soil microbial biomass and activity; ii) the addition of N and P will have interactive effects on soil microbes; iii) the composition of the microbial community will change after N and/or P addition and iv) such changes in microbial communities would feed back on soil carbon pools.

## Materials and Methods

### Ethics statement

No specific permits were required for the described field studies. This research station (Dinghushan Biosphere Reserve) belongs to South China Botanical Garden, Chinese Academy of Sciences. This study also supported by this institute. We confirmed that the location is not privately-owned or protected in any way. We also confirmed that the field studies did not involve endangered or protected species.

### Site description

This study was conducted in the 1200 ha Dinghushan Biosphere Reserve (DHSBR), located in the central part of Guangdong Province, southeastern China (112°10′ E, 23°10′ N). Among the forest types at DHSBR is an old-growth monsoon evergreen broadleaf forest, which is about 250–300 m above sea level and occupies 20% of the reserve. This forest is typical of undisturbed forests of tropical China, having been protected by monks for more than 400 years and experiencing minimal direct human impacts [34]. Major plant species include *Castanopsis chinensis* Hance, *Schima superba* Chardn. & Champ., *Cryptocarya chinensis* (Hance) Hemsl., *Cryptocarya concinna* Hance, *Machilus chinensis* (Champ. Ex Benth.) Hemsl., *Syzygium rehderianum* Merr. & Perry in the tree layer and *Calamus rhabdicladus* Burret, *Ardisia quinquegona* Bl. and *Hemigramma decurrens* (Hook.) Copel in the understory layer [19]. Mean annual litter biomass production is ∼8.3 Mg ha^−1^ yr^−1^ [35]. Stem density, tree height and mean diameter at breast height are summarized in Table 1.

**Table 1:** Indices of the old-growth tropical forest at Dinghushan Biosphere Reserve.

Species	Stem density (tree ha^−1^)	Mean height (m)	Mean diameter at breast height (cm)	Basal area (m^2^ ha^−1^)
*Castanopsis chinensis*	268	9.8	26.0	18.7
*Machilus chinensis*	131	9.0	14.8	4.0
*Schima superba*	185	9.9	18.3	6.4
*Cryptocarya chinensis*	270	8.3	14.3	4.4
*Syzygium rehderianum*	185	8.5	12.9	1.2
Other plants	1587	4.3	4.4	3.5
Total	2625			38.2

Survey was conducted in February 2007 (before the start of N and P fertilization).

The reserve has a typical monsoon and humid climate (*sensu* Holdridge, 1967), with an average annual precipitation of 1927 mm that exhibits a distinct seasonal pattern with 75% falling from March to August and only 6% falling from December to February [36]. The mean annual temperature is 21 °C, with minimum monthly mean temperature of 12.6 °C in January and maximum of 28.0 °C in July; annual mean relative humidity is 80% [36]. Inorganic N deposition measured in throughfall was 33 kg N ha^−1^yr^−1^, with an additional input as dissolved organic N at 15–20 kg N ha^−1^ yr^−1^ [16].

Soil in the reserve is lateritic red earth formed from sandstone [34]. The soil depth in the old-growth forest is more than 60 cm to the top of the C horizon [34]. The forest stands used in the experiment are situated on mountain slopes ranging from 15°–35°. General soil chemical properties are listed in Table 2.

**Table 2:** Soil chemical properties after fertilization treatments, measured in June 2011.

Treatments	Control	N	P	NP	Two-way anova		
					N	P	N*P
pH (H_2_O)	3.69 (0.02) a	3.66 (0.03) a	4.00 (0.03) c	3.86 (0.04) b	**	***	ns
NH_4_^+^-N (mg kg^−1^)	10.7 (1.0) a	9.0 (0.6) a	9.2 (1.3) a	8.1 (0.7) a	ns	ns	ns
NO_3_^−^-N (mg kg^−1^)	3.6 (0.4) a	4.6 (0.7) a	1.9 (0.2) b	3.1 (0.6) ab	*	**	ns
Available P (mg kg^−1^)	2.1 (0.1) a	4.2 (0.8) a	28.0 (2.8) b	21.3 (1.6) c	ns	***	ns
SOC (%)	5. 0(0.3) a	4. 6 (0.3) ab	3.9 (0.2) b	4.5(0.2) ab	ns	*	ns
DOC (mg kg^−1^)	209 (12) a	183 (7) a	185 (7) a	184 (5) a	ns	ns	ns

Notes: SOC (%) and DOC (mg kg^−1^) stand for soil organic carbon and dissolved organic carbon, respectively. Values are means with SE in parentheses (N = 5). Values followed by different letters are significantly different among treatments with *p*<0.05. *, **, *** significance at the level of 0.05, 0.01 and 0.001, respectively.

### Experimental treatment

In 2007, four treatments were established in five replicates each: Control, N-addition (15 g N m^−2^ yr^−1^), P-addition (15 g P m^−2^ yr^−1^), and NP-addition (15 g N m^−2^ yr^−1^ plus 15 g P m^−2^ yr^−1^). Each of the 20 plots were 5 m×5 m were established and surrounded by a 5-m wide buffer strip. Plots size and fertilizer level were similar to those in the experiment in Costa Rica by Cleveland and Townsend [37]. Field plots and treatments were laid out randomly. Applications of N and P were made as NH_4_NO_3_ and NaH_2_PO_4_ solutions sprayed in two monthly portions below the canopy with a backpack sprayer starting from January 2007 and continuing through June 2011. Fertilizer was weighed and mixed with 5 L of water for each plot. Each control plot received 5 L of water without fertilizer.

### Field sampling and measurements

Soil sampling was conducted in June 2011. From each plot, 5 soil cores (2.5 cm inner diameter) were collected randomly from a 10-cm soil depth and combined to one composite sample. The litter layer was carefully removed before sampling. After removing stones and coarse roots, soils were sieved to 2 mm mesh size and divided into two parts, one retained for measuring soil chemical parameters and the other for analysis of microbial biomass and community structure. At the same time, an *in situ* soil-core technique was used to estimate net nitrification rates [38].

Soil moisture content was measured gravimetrically using 10 g of field moist soil sample oven dried at 105 °C for 24 h. Soil pH was measured in a 1∶2.5 soil/water suspension. Soil light-fraction carbon (C) and heavy-fraction C were separated by gravity following the method described by Janzen *et al*. [39] and modified by Izaurralde *et al*. [40]. Soil organic C (SOC) and C content of the light-fraction and heavy-fraction were determined by dichromate oxidation and titration with ferrous ammonium sulfate. Dissolved organic carbon (DOC) in filtered 0.5 M K_2_SO4-extracts of fresh soil sample was measured with a TOC analyser (TOC-VCPH Shimadzu Corp., Japan). NH_4_^+^-N and NO_3_^−^-N in filtered 2 M KCL-extracts of fresh soil sample were measured with a flow injection autoanalyzer (FIA, Lachat Instruments, USA). Available P concentration was analyzed colorimetrically after acidified ammonium persulfate digestion [41].

Soil microbial biomass C (MBC) and microbial biomass N (MBN) were estimated by chloroform fumigation-extraction [42]. Soil respiration was measured using the static chamber and gas chromatography techniques [33]. Soil microbial biomass and community structure was characterized using phospholipid fatty acid (PLFA) analysis as described by Bossio and Scow [43]. The abundance of individual fatty acids was determined as nmol per g of dry soil and standard nomenclature was used [44]. Concentrations of each PLFA were calculated based on the 19∶0 internal standard concentrations. Frostegård and Bååth [45] chose a set of fatty acids to represent bacterial PLFAs, out of which i14∶0, 15∶0, i15∶0, a15∶0, i16∶0, 16∶1ω7c, 17∶0, a17∶0, i17∶0, cy17∶0, 18∶1ω7 and cy19∶0 were present in our samples. We calculated the sum of i14∶0, i15∶0, a15∶0, i16∶0, a17∶0 and i17∶0 as an indicator of gram-positive bacteria. In our study, Gram-negative bacteria were identified by the PLFAs: 16∶1ω7c, cy17∶0, 18∶1ω7 and cy19∶0 [46]. The fungi were identified by the PLFA 18∶2ω6,9c [47], and PLFAs 16∶1ω5c were used as a marker for arbuscular mycorrhizal fungi (AMF) [48]. The actinomycetes were identified by the PLFAs 10Me 16∶0, 10Me 17∶0 and 10Me 18∶0 [49]. Other PLFAs such as 14∶0, 16∶0, 16∶1 2OH, 16∶1ω9c, 17∶1ω8c, 18∶1ω9c, 10Me 19∶0, 18∶3ω6c and 20∶1ω9c were also used to analyze the composition of microbial community. The ratio of 18∶2ω6,9c to total bacterial PLFAs was used to estimate the ratio of fungal to bacterial biomass (F: B) in soils [45], [50].

### Statistical analysis

Two-way ANOVA [Analysis of Variance, PROC GLM from SAS for Windows version 8] was used to examine the difference in soil chemical characteristics, microbial biomass, and F: B ratios among treatments. Twenty-six PLFAs were detected, identified, and included in principal component analysis (PCA) after standardisation for equal unit variance. Redundancy analysis (RDA) was used to test the relationship between soil microbial community (26 PLFAs) and environmental variables. Statistical significance tests for PCA and RDA were run using CANOCO software for Windows 4.5 (Microcomputer Power, Ithaca, NY, USA). Forward selection was based on Monte Carlo permutation (n = 499). Statistically significant differences were identified as P<0.05 unless otherwise stated.

## Results

### Soil chemical properties

**Figure 1:**
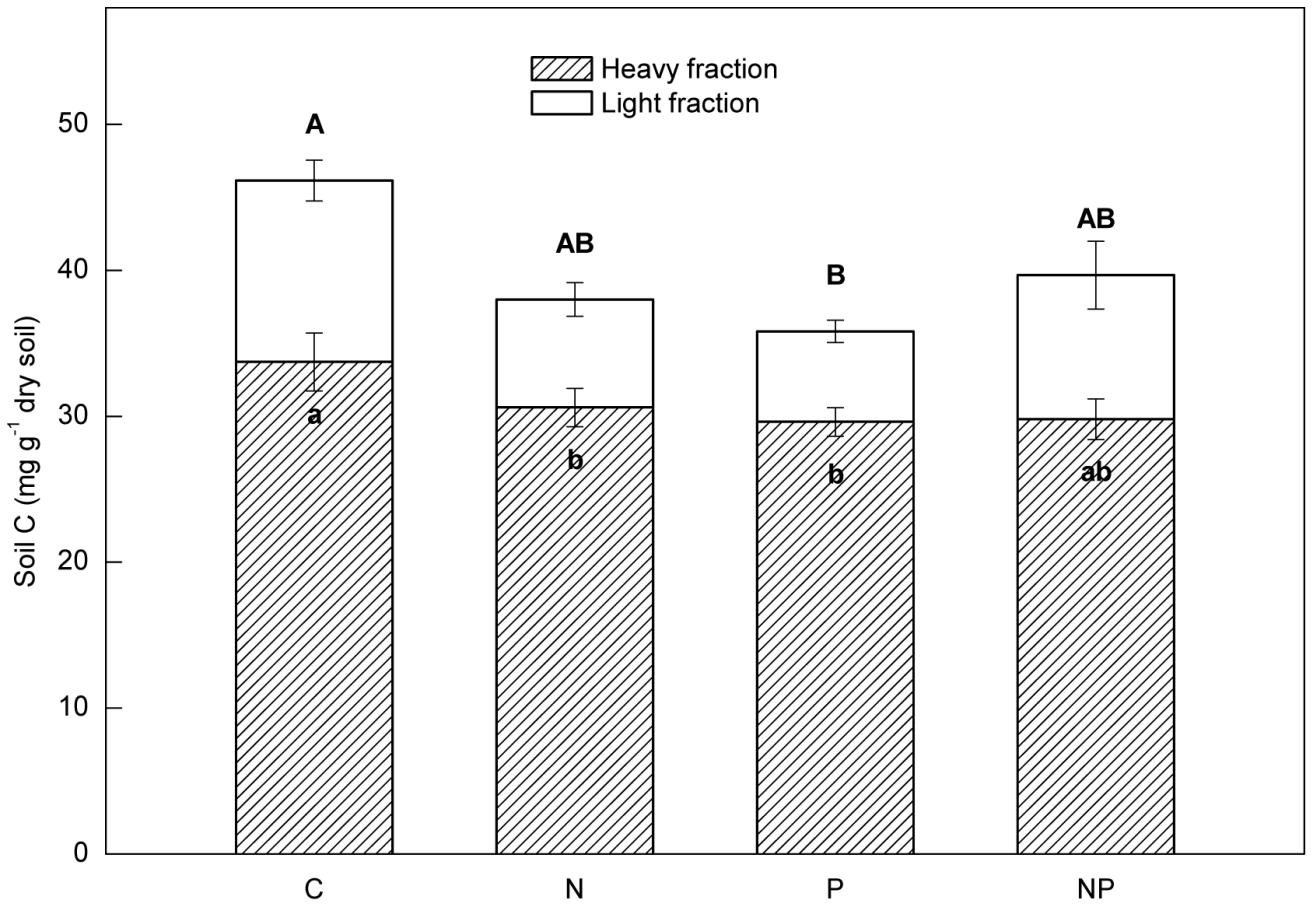
The contribution of two soil fractions to total soil C (Jun. 2011). C: control, N: nitrogen addition, P: phosphorus addition, NP: nitrogen and phosphorus addition. Significant differences (p<0.05) among treatments are indicated by different letters. Error bars show SE (n = 5).

By the time of sampling in June 2011, N and P treatments had significantly altered soil nutrients relative to the control (Table 2). Available P and soil pH were significantly elevated in the P-amended plots. Soil NH_4_^+^ concentrations did not differ among treatments irrespective of the N-addition. Moreover, soil NO_3_^−^ concentrations decreased in the P-addition plots. In addition, SOC decreased significantly in the P-addition plots, but not in the N-addition plots. There were no significant interactions between N and P additions. Light-fraction C in soils was significantly lower in both N-addition and P-addition plots than in the control plots (Fig. 1). Soil nitrification rate was significantly lower in the N-amended plots (1.6 and 0.6 mg N kg^−1^ mo^−1^ in N-addition plots and NP-addition plots) than in the control plots (4.4 mg N kg^−1^ mo^−1^).

### Soil microbial biomass and activity

**Figure 2:**
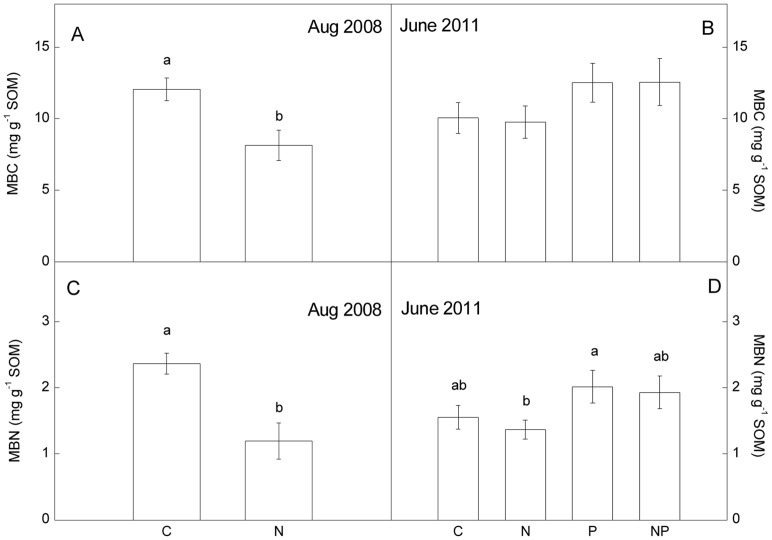
Microbial biomass in soils after nitrogen and phosphorus addition. Data from Aug. 2008 and June 2011. MBC: microbial biomass carbon; MBN: microbial biomass nitrogen. C: control, N: nitrogen addition, P: phosphorus addition, NP: nitrogen and phosphorus addition. Significant differences (p<0.05) among treatments are indicated by different letters. Error bars show SE (n = 5).

**Figure 3:**
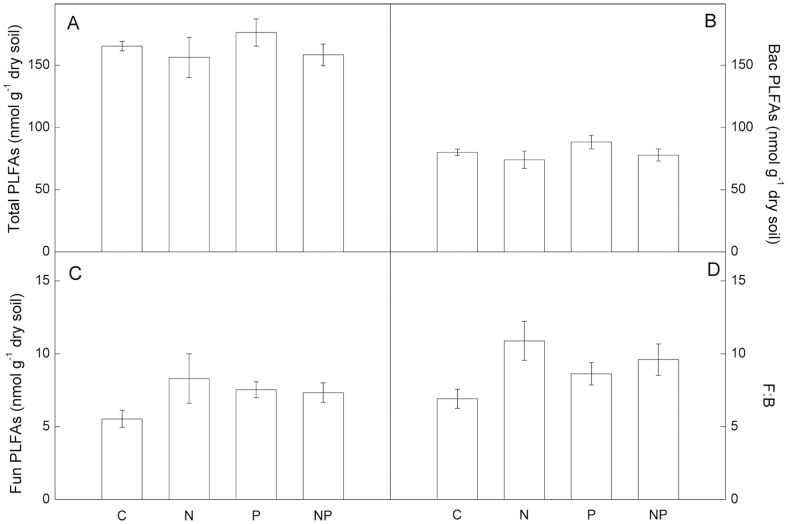
Comparisons of soil microbial PLFAs between treatments. Data from June 2011. C: control, N: nitrogen addition, P: phosphorus addition, NP: nitrogen and phosphorus addition. Bac PLFAs: Bacterial PLFAs; Fun PLFAs: Fungal PLFAs; F:B: the ratio of fungal to bacterial PLFAs. Significant differences (p<0.05) among treatments are indicated by different letters. Error bars show SE (n = 5).

**Figure 4:**
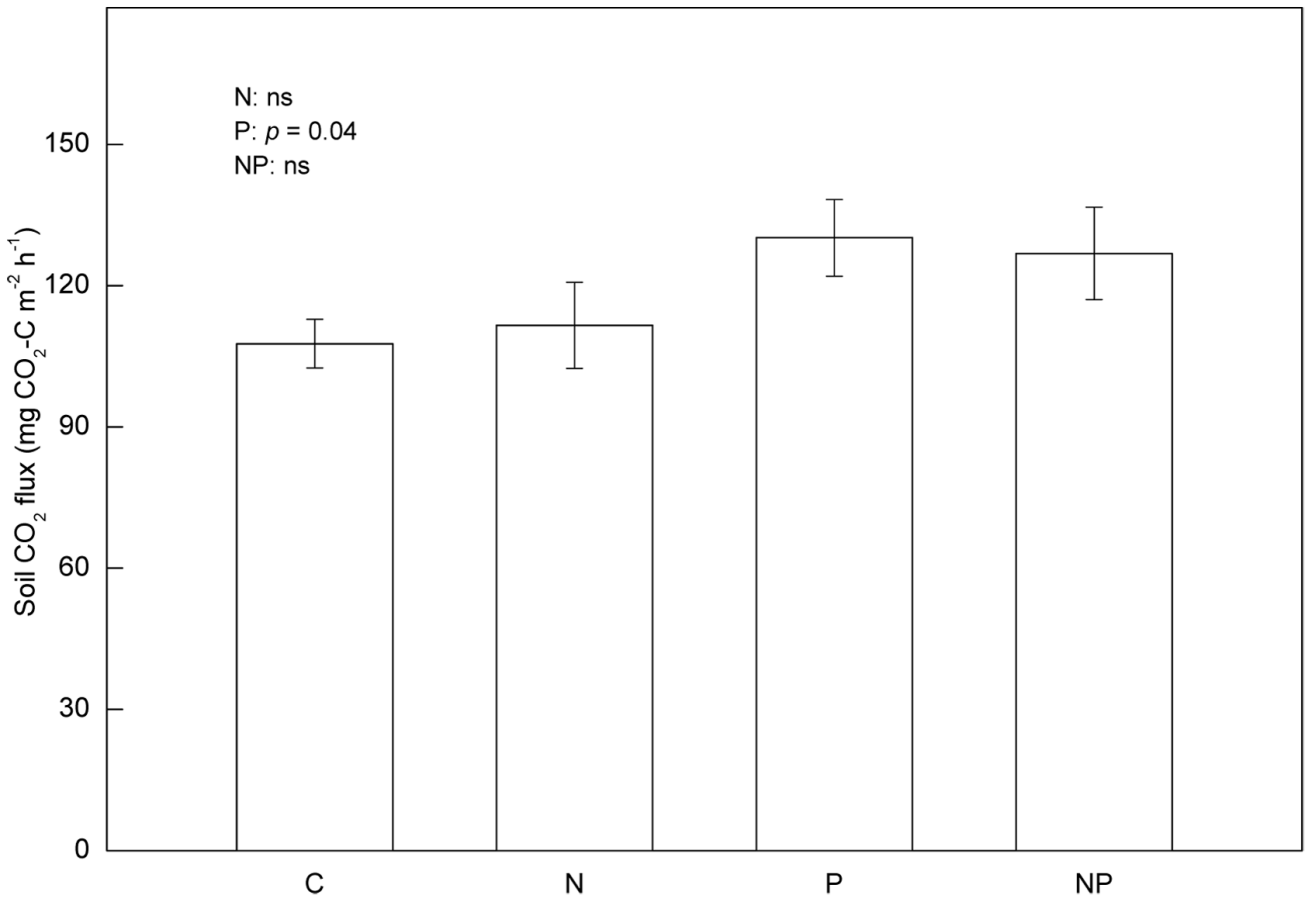
Comparisons of soil respiration between treatments. Values are means for three months. (Data from May-July 2011). C: control, N: nitrogen addition, P: phosphorus addition, NP: nitrogen and phosphorus addition. Error bars show SE (n = 5).

Although both MBC and MBN decreased significantly by N addition after 18-month, there were no significant differences in MBC between treatments over the long-term, 52-month period (Fig. 2). Moreover, there were no interactions between N addition and P addition on soil microbial biomass (Appendix S1). Total microbial biomass, bacterial biomass and fungal biomass were also not significantly different among treatments (Fig. 3). However, MBN was significantly lower in the N-addition plots than in the P-addition plots in June 2011 (Fig. 2). Fertilization with P significantly increased soil respiration, and there was no effect of N fertilization (Fig. 4).

### Soil microbial community structure

**Figure 5:**
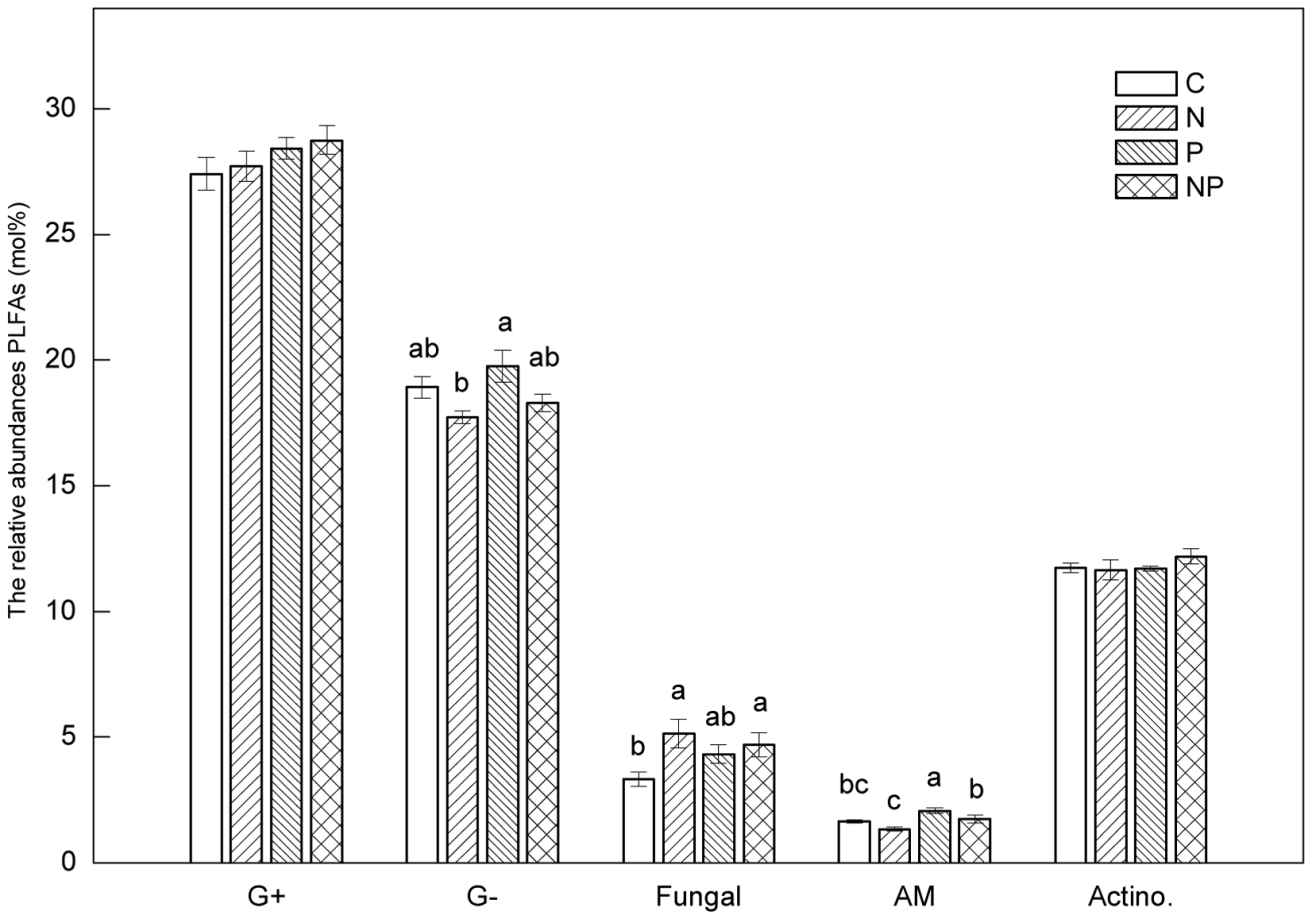
The relative abundances of the individual PLFAs (mol %) in soil samples. G^+^: the proportion of gram-positive bacterial PLFAs; G^−^: the proportion of gram-negative bacterial PLFAs; Fungi: the proportion of fungal PLFAs; AM: the proportion of AM fungal PLFAs; Actino.: the proportion of actinomycetes PLFAs. Significant differences (p<0.05) among treatments are indicated by different letters. Error bars show SE (n = 5).

Mean abundances of gram-negative bacteria and arbuscular mycorrhizal (AM) PLFAs were significantly higher in the P-addition plots, whereas the mean abundances of fungal PLFAs was significantly lower in the control plots compared with the N-amended plots (Fig. 5). Fungal: bacterial ratios were also significantly lower in the control plots than in the N-addition plots (Fig. 3). Mean abundance of gram-positive bacteria and actinomycetes PLFAs did not differ among treatments, although relative abundances of individual PLFA 10Me 16∶0, 10Me 17∶0, and 10Me 18∶0 (all actinomycete markers) were significantly different among treatments. Relative abundance of 10Me 17∶0 was higher in the N-addition plots than in the P-addition plots, whereas 10Me 18∶0 was lower in the N-addition plots compared with the P-addition plots. Relative abundance of 10Me 16∶0 was higher in the NP-addition plots than in the P-addition plots (Fig. 5).

The first two axes produced by principal components analysis accounted for 63.6% of the total variation in the PLFA profile, with PC1 explaining 37.1% of the variation and separating the P addition versus non-P addition plots (P<0.001, Appendix S1, S2). Loadings of individual PLFAs showed that the samples in the P-addition plots were characterized mainly by high concentrations of the monounsaturated PLFAs 16∶1w7c, 16∶1w9c, 18∶1w7c (indicated Gram-negative bacteria), and 16∶1w5c (indicated AM fungi) (Appendix S3). These results were also confirmed by PLFA relative abundance data.

The correlations between soil microbial community and soil chemical properties revealed that six soil variables, including pH, soil organic carbon (SOC), NO_3_^−^, NH_4_^+^, available P, soil moisture content (SMC), explained 45.4% of the variation in soil microbial community composition. Soil microbial community structure was significantly related to NO_3_^−^, available P and SOC, with axis 1 explaining 29.3% of the variance and axis 2 explaining 16.1% (Appendix S4).

## Discussion

### Soil microbial biomass and community structure

#### Effects of N addition

Nitrogen additions decreased soil microbial biomass in the short term but returned to pre-treatment levels over the long term. This was an unexpected effect of long term N addition and was likely due to microbial populations adapting to the higher N conditions created by 52 months of N additions. One possible cause of decreased microbial biomass C is soil acidification resulting from NH_4_^+^ uptake by plants, nitrification of NH_4_^+^ in soils, and NO_3_^−^ leaching [51]. In the present study, soil pH was already very low in the control plots and there was no significant difference between control and long term N addition plots (Table 2), suggesting that soil pH may not be affected by N addition under this N-saturated system. Balser (2001) also found that long term N addition had no effect on microbial biomass in three Hawaiian forest soils [52]. In contrast, N addition in temperate forests typically increases microbial biomass at a short term basis [53] but over the longer term, biomass generally decreases [51], [54]–[56].

Unlike soil microbial biomass, effects of experimental inputs of N to forests on soil microbial communities are inconsistent in the literature. Nilsson *et al*. (2007) reported that N input had no effect on total fungal biomass in the soils of oak forests along a natural N deposition gradient [57]. Several studies have shown that, fungal biomarkers were reduced in soils of N-fertilized plots [58], [59]. In contrast, increases in fungal biomarkers in N manipulation experiments have been reported in north temperate forest soils [60]. A common element to these studies is that they were carried out in temperate forests. Less is known, however, about how N deposition alters microbial community in tropical regions. In the present study, although there was no effect of long term N addition on soil microbial biomass, microbial community structure contrasted notably in the control versus N-addition plots, largely due to N-mediated increases in relative abundance of fungal PLFAs. Consequently, F: B ratios were significant higher in the N-addition plots comparing to the control plots (Figs.3–5). This is consistent with results reported by Balser (2001), which showed that microbial community composition was affected by long term N addition in three Hawaiian forest soils [52].

The induced changes in microbial communities was also supported by the fact that Gram negative bacteria (e.g., 16∶1w7 and 18∶1w7), which include several nitrifying species, decreased significantly in N-addition plots compared to the control plots, corresponding to lower net nitrification in the N-addition plots comparing to the control plots (Figs. 4,5). Gilliam *et al*. (2011) found that the Gram-negative PLFA 18∶1n7c (Gram negative bacteria) was predominant in soils with highest rates of net nitrification soils at Fernow Experimental Forest, a central Appalachian hardwood forest of West Virginia, USA [26]. Billings and Ziegler (2008) observed an increase in the activity of Gram negative bacteria for pine forest soil fertilized with NH_4_NO_3_ [61]. Notably, N addition actually decreased net nitrification and relative abundance PLFA 16∶1w7c and 18∶1w7c, which include NH_4_-oxidizing and NO_2_-oxidizing Gram negative bacteria.

#### Effects of P addition

In contrast to effects of N, addition of P increased soil microbial biomass, indicating that P availability is a limiting factor for microbial growth in this old-growth tropical forest. This stimulating effect of P on microbial biomass, however, was also transient [33] (Fig. 2). Soil respiration was significantly increased after long-term P addition (Fig 4), suggesting that microbial activity is higher in the presence of addition P, allowing for more rapid transformation of soil organic matter. In a previous study, we found a similar response, concluding that it arose from enhanced P-mediated increases in C availability to microbes in soils of this forest [33], thus constraining the microbial processes that relate to SOM processing.

Addition of P altered microbial community structure by significantly increasing the relative abundance of AM fungi, which obtain C from their host plants in return for mineral nutrients [62]. With N or P limiting to plant growth, plant will invest more C to AM fungi in exchange for nutrients [63]. Conversely, if N or P availability rises, a decline in AM abundance is expected [64]. However, our result is inconsistent with the expectation that P abundance suppresses plant investment in the mycorrhizal symbiosis. One possible reason was that AM fungi were initially nutrient-limited. Because this old-growth forest is N saturated and with low P availability. P additions may increase mycorrhizal growth. Since mycorrhizal fungi are more efficient scavengers for nutrients from the soil than are plant roots, the threshold for nutrient limitation may be lower for mycorrhizal fungi than for plants [65], [66]. Another possible reason was increased in soil pH after P addition, as this is often associated with increased AM biomass in soil (PLFA 16∶1ω5c) [67], [68].

Several studies also reported that apatite addition had a significant positive influence on fungal growth in P-poor forests, but not in forests with sufficient P [69], [70]. However, Groffman and Fisk (2011) found that P addition had no effect on microbial biomass and activity in a northern hardwood forest (Hubbard Brook Experimental Forest, New Hampshire, USA), concluding that P did not limit microbial biomass and respiration [71]. In contrast, the whole-watershed Ca treatment at Hubbard Brook enhanced AM colonization [72]. Kaspari *et al*. (2008) and Wright *et al*. (2011) have reported that K-limitation of microbial decomposition and N, P and K all limit a suite of ecosystem processes in a tropical forest in Panama [13], [73]. These studies reveal that tropical forests may possess a greater complexity of multiple nutrient limitations than what has previously been considered.

#### Interactive effects of N and P addition

We expected that N and P additions would have negative and positive effects, respectively, on microbial biomass, and that microbial community composition would vary with fertilization. Furthermore, as interactive effects of combined N and P enrichment are common in many terrestrial ecosystems [1], we expected that simultaneous N and P addition would produces notable interactive effects on soil microbes in this tropical forest; to the contrary, however, none were found (Appendix S1). We suggest that this occurs because this old-growth forest is N saturated, exhibiting net loss of 8–16 kg N ha^−1^yr^−1^ from the soil [3], [16]. Thus, P rather than N is limiting factor for microbe growth.

### Soil organic carbon

Nutrient enrichments may feed back on ecosystem C through the effects on microbial biomass and community composition. Microbial biomass, which represents an important labile pool of nutrients in soil, plays a significant role in nutrient cycling in ecosystems [74]. Changes in the size of the microbial biomass pool may indicate changes in the soil organic matter pool. Reciprocally, microbial biomass also depends on soil organic matter [75]. In the present study, soil microbial biomass C was not significantly different among treatments (Fig. 3), suggesting that microbial biomass was not responsible for the N- and P-induced changes in soil C pool in this tropical forest.

However, we found that soil microbial community structure was significantly different among treatments. N addition increased fungal abundance, with detectable decreases in labile C compounds in this tropical forest. Previous results at this site have shown that experimental N addition significantly decreased litter decomposition [22], [24]. Several studies in temperate systems have demonstrated that fertilization with N may stimulate decay of more labile C, suppress degradation of lignin, and stimulate formation of recalcitrant material [76]–[78]. Cusack *et al*. (2011) reported that N fertilization reduced C content in the light fraction and increased C content in the heavy fraction in two tropical forests [79]. Our results support this conclusion in part: N addition had a negative effect on C content in the light fraction, but not in the heavy fraction. This finding may be related to the higher relative abundance of fungal PLFAs in N-addition plots compared to control plots (Figs. 3–5). However, Ramirez *et al*. (2012) found that N addition depresses soil microbial activity by shifting soil bacterial communities, yielding communities that are less capable of decomposing more recalcitrant soil carbon pools [30]. Strickland *et al*. (2009) observed no relationship between microbial community structure and C mineralization processes in soil [80]. Those studies indicated that C cycling is changing in response to N addition, driven by complex interactions among microbial composition, enzymatic capability and soil C chemistry.

Cleveland *et al*. (2002) reported that in ferralsols of tropical rainforests microbial C use was strongly constrained by P availability and suggested that more P-poor forests may display longer-term soil storage of novel inputs of C [29]. Our results that light fraction SOM and SOC significantly decreased after P-addition support this suggestion. Zhou *et al*. (2006) reported an accumulation of soil C (0–20 cm depth) at about 54 g C m-2 yr-1 over two decades in the old-growth forest [21]. One of the possible reasons is that the P-limited microbial community may increase the rates of belowground C storage [33]. Results from our study further indicated that a P-limited microbial community may actually help retain more labile forms of C in these tropical forests.

Our results have demonstrated that both the microbial community and chemistry of the SOM pool can vary greatly in response to N versus P additions. Although C content in the light fraction decreased significantly for both treatments, total soil C decreased significantly only in the P-addition plots, suggesting that soil C changes induced by fertilization may be more sensitive to P addition than to N addition in these tropical forests. Not only was soil microbial community structure significantly related to NO_3_^−^, available P, and SOC (Appendix S4), but soil NO_3_^−^ concentrations significantly decreased and soil available P increased in the P-addition plots (Table 2). Previous studies have shown that litterfall production was not affected by N addition, but increased by P addition, supporting the idea that P rather than N availability may be a limiting factor for plant growth, such that P addition may increase the supply of labile plant C to microbes [23], [33]. At the same time, we found that AM fungal abundance was significantly increased in the P-addition plots compared to the N-addition plots. This result indicated that abundance of AM fungi may play a larger role in organic matter decomposition and C cycling.

## Supporting Information

Appendix S1 **Effects of N addition, P addition and two-way interactions of N addition and P addition on soil properties and microbial characteristics.**
(DOC)

Appendix S2 **The relative abundances of the individual PLFAs (mol %) in soil samples.**
(DOC)

Appendix S3 **The phospholipid fatty acid (PLFA) pattern in soil samples.**
(DOC)

Appendix S4 **Redundancy analysis of PLFA profiles used 26 PLFAs as species and six environmental parameters.**
(DOC)
