# Supplementary material for: Interactive Effects of Nitrogen and Phosphorus on Soil Microbial Communities in a Tropical Forest
Source: PLoS One. 2013 Apr 12;8(4):e61188. doi: 10.1371/journal.pone.0061188 (PMC3625167; doi:10.1371/journal.pone.0061188)
Supplement: Appendix S1 — Effects of N addition, P addition and two-way interactions of N addition and P addition on soil properties and microbial characteristics. (DOC) [file pone.0061188.s001.doc]

**Appendix S1.** Effects of N addition, P addition and two-way interactions of N addition and P addition on soil properties and microbial characteristics, n = 5.

| Factors | N additions | | P additions | | N*P | |
| --- | --- | --- | --- | --- | --- | --- |
|  | *F* | *P* | *F* | *P* | *F* | *P* |
| Light C | 0.19 | 0.67 | 1.50 | 0.24 | **8.16** | **0.01** |
| Heavy C | 1.01 | 0.33 | 2.83 | 0.11 | 1.26 | 0.28 |
| MBC | 0.01 | 0.94 | 3.96 | 0.06 | 0.02 | 0.90 |
| MBN | 0.44 | 0.52 | **5.97** | **0.03** | 0.06 | 0.81 |
| Total PLFAs | 1.54 | 0.23 | 0.36 | 0.56 | 0.17 | 0.69 |
| Bacterial PLFAs | 2.53 | 0.13 | 1.36 | 0.26 | 0.19 | 0.67 |
| Fungal PLFAs | 1.67 | 0.21 | 0.27 | 0.61 | 2.21 | 0.16 |
| F : B | **6.12** | **0.02** | 0.05 | 0.83 | 2.25 | 0.15 |
| G+ (mol %) | 0.31 | 0.59 | 3.18 | 0.09 | 0 | 0.99 |
| G- (mol %) | **9.01** | **0.009** | 2.55 | 0.13 | 0.09 | 0.77 |
| Fun (mol %) | **6.27** | **0.02** | 0.37 | 0.55 | 2.73 | 0.12 |
| AM (mol% ) | **8.05** | **0.01** | **14.16** | **0.002** | 0.03 | 0.87 |
| Actino. (mol% ) | 0.5 | 0.49 | 0.88 | 0.36 | 1.08 | 0.31 |
| i14:0 | 0.26 | 0.62 | 1.34 | 0.26 | 0.40 | 0.54 |
| i15:0 | 0.47 | 0.50 | **7.29** | **0.02** | 0.01 | 0.92 |
| a15:0 | 0.13 | 0.73 | **9.67** | **0.007** | 1.33 | 0.27 |
| i 16:0 | 2.61 | 0.13 | 3.74 | 0.07 | 0.02 | 0.88 |
| i 17:0 | 0 | 0.97 | **4.33** | **0.05** | 0.01 | 0.93 |
| a 17:0 | 0.26 | 0.62 | **4.94** | **0.04** | 0.68 | 0.42 |
| 16:1 w9c | **5.96** | **0.03** | **39.7** | **< 0.001** | **12.43** | **0.003** |
| 16:1 w7c | **9.73** | **0.007** | **23.53** | **< 0.001** | 0.05 | 0.83 |
| cy 17:0 | **7.32** | **0.02** | **5.73** | **0.03** | 1.56 | 0.23 |
| 18:1 w7 | **22.73** | **< 0.001** | **5.68** | **0.03** | 1.62 | 0.22 |
| cy19:0 | 0.1 | 0.76 | 1.33 | 0.27 | 0.28 | 0.60 |
| 15:0 | 2.93 | 0.11 | 0.80 | 0.38 | 1.30 | 0.27 |
| 17:0 | 0.42 | 0.53 | 2.43 | 0.14 | 0.98 | 0.34 |
| 18:0 | 3.29 | 0.09 | 2.63 | 0.12 | 1.02 | 0.33 |
| 10Me 16:0 | 1.69 | 0.21 | 1.40 | 0.25 | 3.16 | 0.09 |
| 10Me 17:0 | 3.15 | 0.10 | **5.37** | **0.03** | 0.13 | 0.72 |
| 10Me 18:0 | 2.85 | 0.11 | 1.79 | 0.20 | 0.34 | 0.57 |
| 18:1 w9c | 0 | 0.99 | 0.10 | 0.75 | 0.14 | 0.72 |
| PC1 | 2.96 | 0.10 | **21.2** | **< 0.001** | 0.18 | 0.68 |
| PC2 | 0.19 | 0.67 | 0.02 | 0.88 | 0.05 | 0.82 |

**Note:** N * P: interactions between N addition and P addition; Light C: light fraction of carbon; Heavy C: heavy fraction of carbon; MBC: microbial biomass carbon; MBN: microbial biomass nitrogen; F: B indicates the ratio of fungal to bacterial PLFAs. G^+^: the proportion of gram-positive bacterial PLFAs; G^-^: the proportion of gram-negative bacterial PLFAs; Fungi: the proportion of fungal PLFAs; AM: the proportion of AM fungal PLFAs; Actino.: the proportion of actinomycetes PLFAs. Results are from two-way factorial ANOVA for the soil property and microorganism variables.
