# Supplementary material for: Interactive Effects of Nitrogen and Phosphorus on Soil Microbial Communities in a Tropical Forest
Source: PLoS One. 2013 Apr 12;8(4):e61188. doi: 10.1371/journal.pone.0061188 (PMC3625167; doi:10.1371/journal.pone.0061188)
Supplement: Appendix S2 — The relative abundances of the individual PLFAs (mol %) in soil samples. (DOC) [file pone.0061188.s002.doc]

**Appendix S2.** The relative abundances of the individual PLFAs (mol %) in soil samples. C: control, N: nitrogen addition, P: phosphorus addition, NP: nitrogen and phosphorus addition. Significant differences (p< 0.05) among treatments are indicated by asterisk. Error bars show SE (n = 5).
