# Supplementary material for: Interactive Effects of Nitrogen and Phosphorus on Soil Microbial Communities in a Tropical Forest
Source: PLoS One. 2013 Apr 12;8(4):e61188. doi: 10.1371/journal.pone.0061188 (PMC3625167; doi:10.1371/journal.pone.0061188)
Supplement: Appendix S3 — The phospholipid fatty acid (PLFA) pattern in soil samples. (DOC) [file pone.0061188.s003.doc]

**Appendix S3.** The phospholipid fatty acid (PLFA) pattern in soil samples (Jun. 2011). PLFA data were subjected to principal component analysis (PCA). C: control, N: nitrogen addition, P: phosphorus addition, NP: nitrogen and phosphorus addition.
