# Supplementary material for: Interactive Effects of Nitrogen and Phosphorus on Soil Microbial Communities in a Tropical Forest
Source: PLoS One. 2013 Apr 12;8(4):e61188. doi: 10.1371/journal.pone.0061188 (PMC3625167; doi:10.1371/journal.pone.0061188)
Supplement: Appendix S4 — Redundancy analysis of PLFA profiles used 26 PLFAs as species and six environmental parameters. (DOC) [file pone.0061188.s004.doc]

**Appendix S4.** Redundancy analysis of PLFA profiles used 26 PLFAs as species and six environmental parameters. Vectors represent environmental variables. SOC, soil organic carbon; Avai P, available phosphorus; SMC, soil moisture content. *Squares* control, *triangles* N-addition, *circles* P-addition, *diamond* NP-addition.
